# Frequency, prognostic impact, and subtype association of 8p12, 8q24, 11q13, 12p13, 17q12, and 20q13 amplifications in breast cancers

**DOI:** 10.1186/1471-2407-6-245

**Published:** 2006-10-13

**Authors:** Anne Letessier, Fabrice Sircoulomb, Christophe Ginestier, Nathalie Cervera, Florence Monville, Véronique Gelsi-Boyer, Benjamin Esterni, Jeannine Geneix, Pascal Finetti, Christophe Zemmour, Patrice Viens, Emmanuelle Charafe-Jauffret, Jocelyne Jacquemier, Daniel Birnbaum, Max Chaffanet

**Affiliations:** 1 Centre de Recherche en Cancérologie de Marseille, Département d'Oncologie Moléculaire, UMR599 Inserm/Institut Paoli-Calmettes, Marseille, France; 2 Centre de Recherche en Cancérologie de Marseille, Département de BioPathologie, UMR599 Inserm/Institut Paoli-Calmettes, Marseille, France; 3 Faculté de Médecine, Université de la Méditerranée, Marseille, France; 4 Centre de Recherche en Cancérologie de Marseille, Département d'Oncologie Médicale, UMR599 Inserm/Institut Paoli-Calmettes, Marseille, France

## Abstract

**Background:**

Oncogene amplification and overexpression occur in tumor cells. Amplification status may provide diagnostic and prognostic information and may lead to new treatment strategies. Chromosomal regions 8p12, 8q24, 11q13, 17q12 and 20q13 are recurrently amplified in breast cancers.

**Methods:**

To assess the frequencies and clinical impact of amplifications, we analyzed 547 invasive breast tumors organized in a tissue microarray (TMA) by fluorescence in situ hybridization (FISH) and calculated correlations with histoclinical features and prognosis. BAC probes were designed for: (i) two 8p12 subregions centered on *RAB11FIP1 *and *FGFR1 *loci, respectively; (ii) 11q13 region centered on *CCND1*; (iii) 12p13 region spanning *NOL1*; and (iv) three 20q13 subregions centered on *MYBL2, ZNF217 *and *AURKA*, respectively. Regions 8q24 and 17q12 were analyzed with *MYC *and *ERBB2 *commercial probes, respectively.

**Results:**

We observed amplification of 8p12 (amplified at *RAB11FIP1 *and/or *FGFR1*) in 22.8%, 8q24 in 6.1%, 11q13 in 19.6%, 12p13 in 4.1%, 17q12 in 9.9%, 20q13^Z ^(amplified at *ZNF217 *only) in 9.9%, and 20q13^Co ^(co-amplification of two or three 20q13 loci) in 8.5% of cases. The 8q24, 12p13, and 17q12 amplifications were correlated with high grade. The most frequent single amplifications were 8p12 (9.8%), 8q24 (3.3%) and 12p13 (3.3%), 20q13^Z ^and 20q13^Co ^(1.6%) regions. The 17q12 and 11q13 regions were never found amplified alone. The most frequent co-amplification was 8p12/11q13. Amplifications of 8p12 and 17q12 were associated with poor outcome. Amplification of 12p13 was associated with basal molecular subtype.

**Conclusion:**

Our results establish the frequencies, prognostic impacts and subtype associations of various amplifications and co-amplifications in breast cancers.

## Background

Amplification is a frequent and important mechanism for oncogene overexpression in breast tumor cells. Several amplified regions may participate to breast tumor initiation and/or progression [1]. Chromosomal regions 8p12, 8q24, 11q13, 17q12 and 20q13 are amplified in a consistent proportion of breast tumors [2-5]. The 12p13 region was also found amplified in some breast tumors [6,7].

Amplification status may be determined in clinics as an indicator of prognosis or before applying a specific treatment: *ERBB2 *amplification, found in 15 to 25% of breast cancers, is a marker of adverse prognosis and encodes a tyrosine kinase receptor that is the target of trastuzumab (Herceptin) [8-10]. Amplifications at 8p12, 8q24, 11q13, 12p13, and 20q13 have also potential clinical interest as prognosis markers and/or therapeutic targets. Amplification of the 8p12 region is found in around 15% of breast cancers [11-13]. Although the identity of the driver genes has not been definitely established, the importance of the *FGFR1 *tyrosine kinase receptor gene has been suggested [5,14]. Amplification of this region has an adverse impact on prognosis in breast cancer [5]. *MYC *is localized in 8q24 and encodes a nuclear protein that plays a role in cell cycle progression. Amplification of 8q24 occurs in up to 20% of breast cancers and is associated with a poor clinical outcome [15-21]. *CCND1 *localized in 11q13 encodes cyclin D1, which is active during the G1 phase of the cell cycle. Amplification of *CCND1 *occurs in 10 to 30% of breast cancers [3,22-24]. Amplification of 12p13 is not a frequent event in breast cancers but genomic studies indicate its potential importance in basal breast cancers [7,25]. The 20q13 chromosomal region is amplified in 5% to 20% in breast tumors and its prognosis impact is unclear [26-29]. Several potential oncogenes have been suggested, including *MYBL2 *[23,30], *AURKA *[31], and *ZNF217 *[32-34].

To assess the frequencies and to evaluate the impact on prognosis of amplifications and co-amplifications, we analyzed 547 breast tumors organized in a tissue microarray (TMA) by fluorescence in situ hybridization (FISH) with probes covering amplified regions. BAC probes were designed for: (i) two 8p12 subregions [5] centered on *RAB11FIP1 *and *FGFR1 *loci, respectively; (ii) 11q13 region centered on *CCND1*; (iii) 12p13 region spanning *NOL1*; and (iv) three 20q13 subregions [29] centered on *MYBL2, ZNF217 *and *AURKA*, respectively. Regions 8q24 and 17q12 were analyzed with *MYC *and *ERBB2 *commercial probes, respectively.

## Methods

### Patients and histological samples

We studied a consecutive series of 547 unilateral localized invasive breast carcinomas from women treated at the Institut Paoli-Calmettes between October 1987 and December 1999. According to the WHO classification, this series comprised 386 ductal, 72 lobular, 37 tubular, 8 medullary carcinomas and 44 other histological types. They were obtained after informed consent and stored in an anonymous fashion according to an approval of the local Ethics Committee. The Ethics Committee of the Institut Paoli-Calmettes (Marseille's Cancer Institute) approved the use of these specimens and the data in research. The average age at diagnosis was 59 years (range 25–94). Raw survival data were either obtained from the cancer registry of the Institut Paoli-Calmettes or collected from the patients attending physicians. The pathologic stage, tumor diameter, and nodal status were obtained from the primary pathology reports. A total of 254 tumors were associated with lymph node invasion and 403 were positive for estrogen receptor. All slides from all tumors were reviewed by one of two pathologists (J. J. and E.C.J.) to define the various histoclinical factors collected for this series. They included patient age, invasive histological type, pathological tumor size, Scarff-Bloom-Richardson (SBR) grade (I to III), peritumoral vascular invasion, axillary lymph node status, estrogen receptor expression (ER), progesterone receptor expression (PR), P53 status, as evaluated by immunohistochemistry (IHC) with a positivity cut-off value of 1%, ERBB2 status, evaluated by IHC with the 0-3+ score as illustrated by the HercepTest kit scoring guidelines (DakoCytomation, Coppenhagen, Denmark), and Ki67 status as evaluated by IHC with a positive cut-off value at 20%. This study was approved and executed in compliance with our institutional review board.

### Tissue microarray construction

Tissue microarray (TMA) was prepared as described previously [35]. Five-μm sections of the resulting TMA block were made and used for fluorescence in situ hybridization (FISH) and IHC analysis after transfer onto glass slides.

### Fluorescence In Situ Hybridization on TMA analysis

To characterize the 8q24 and 17q12 amplified regions, FISH on TMA was done according to the histology FISH instructions of DakoCytomation with the *MYC *probe and *HER2 *FISH pharmDx™ kit (DakoCytomation, Coppenhagen, Denmark). To characterize the 8p12, 11q13, 12p13, and 20q13 amplified regions, FISH on TMA was done according to published protocols [36,37]. Two 8p12 subregions centered on *RAB11FIP1 *and *FGFR1 *loci, respectively, were analyzed. From telomere to centromere, the different BAC pools were constituted as follows: BAC pool 1 (*RAB11FIP1 *region): RP11-863K10 (AC138356; chr8:37,630,477-37,820,753), RP11-113G10 (chr8:37,715,876-37,881,776), RP11-457O21 (chr8:37,881,796-38,044,340); BAC pool 2 (*FGFR1 *region): RP11-90P5 (AC084024.17; chr8:38,091,019-38,226,422), RP11-513D5 (AC087362.13; chr8:38,216,677-38,358,846), RP11-100B16 (chr8:38,358,839-38,522,417), RP11-675F6 (AC069120.9; chr8:38,485,749-38,646,922). The 11q13 region centred on *CCND1 *was analyzed with the following combination of BAC pools, from centromere to telomere: RP11-300I6 (AP001888; chr11:69,162,462-69,323,966), RP11-643C9 (chr11:69,297,662-69,494,887), RP11-626H12 (AP003555; chr11:69,478,620-69,600,219). The 12p13 region spanning *NOL1 *region was analyzed with, from telomere to centromere, RP5-940J5 (AC006064; chr12:6,422,311-6,594,917), RP11-433J6 (AC135892; chr12:6,579,330-6,755,900), RP11-578M14 (chr12:6,699,832-6,884,088) BAC pool. Three 20q13 subregions of amplification corresponding to *MYBL2, ZNF217 *and *AURKA *loci were analyzed with locus-specific BAC pools, from centromere to telomere : *MYBL2 *locus BAC pool : RP11-69I10 (chr20:41,467,083-41,631,733), RP11-153L9 (chr20:41,659,456-41,808,516), RP5-1030M6 (AL035089; chr20:41,816,168-41,989,971); *ZNF217 *locus BAC pool : RP11-91L1 (chr20:51,421,217-51,572,829), RP4-724E16 (AL157838; chr20:51,561,511-51,690,363), RP11-299C12 (chr20:51,647,272-51,837,964); and *AURKA *locus BAC pool : RP11-380D15 (AL139824; chr20:54,122,911-54,316,054), RP5-1167H4 (AL121914; chr20:54,336,458-54,472,150), RP5-1153D9 (AL109806; chr20:54,472,051-54,566,171). Genomic information was taken from the UCSC Genome Browser on Human (March 2006 Assembly), which is based on NCBI Build 35 (National Center for Biotechnology Information, U.S. National Library of Medicine 8600 Rockville Pike, Bethesda, MD, USA).

DNA from BAC clones were purified, labeled and individually verified for their specificity of their addressed regions. All BAC clones were obtained from the BACPAC resource (Children's Hospital Oakland – BACPAC Resources, Oakland, CA, USA). After counterstaining with Vectashield containing 4,6-diamidino-2-phenylindole (DAPI) (Vector, Burlingame, CA, USA), images were analyzed with a microscope (DMRXA, Leica Microsystèmes, Marseille, France), captured with a CCD camera, filtered and processed with ISIS software (In Situ Imaging Systems, Metasystems Hard- und Software GmbH, Altlussheim, Germany)[38].

Fluorescence was scored on a minimum of 50 nuclei per tumor. The 50 nuclei of cancer cells were representative of the overall cell heterogeneity of the tumor. For each region analyzed, two observers read the TMA independently. The region analyzed was considered as amplified when the number of BAC pool signal was >5 in the cell. Tumors were defined as amplified when 10% or more of tumor cells showed such amplification.

### Immunohistochemistry analysis

The characteristics of the antibodies used are listed in Table 1. IHC was done as previously described [35], using LSAB2 kit in the autostainer (Dako Autostainer, Copenhagen, Denmark). Results were evaluated under a light microscope by two pathologists (EC-J, JJ) and scored by the quick score (QS) as previously done [35], except for ERBB2 status, which was evaluated with the Dako scale (HercepTest kit scoring guidelines). For each tumor, the mean of the score of a minimum of two core biopsies was calculated.

### Statistical methods

Amplification data were summarized by frequencies and percentages. Clinical data were dichotomized as follows: Amplicon: amplified vs. non-amplified, grade: I vs. II/III, age: <50 vs. ≥ 50, tumor size: pT1 vs. pT2/pT3, peritumoral vascular invasion: absent vs. present, estrogen receptor and progesterone receptor: negative vs. positive, Ki67: <20 vs. ≥ 20 and axillary lymph node: negative vs. positive.

The association between two categorical variables was examined using Fisher's exact or χ^2 ^tests. The primary endpoint was the metastasis-free survival (MFS), which was defined by the time interval between the diagnosis of breast cancer and a distant metastasis. Metastasis-free patients were right censored at the date of the last follow-up, death, recurrence of local or regional disease, or development of a second primary cancer. Survival curves were derived from Kaplan-Meier estimates and compared by log-rank test. Significant changes of relative risks of metastasis according to the amplification status were explored using Cox's proportional hazard models in univariate and multivariate analysis. Multivariate models were built using a backward stepwise selection of variables to minimize the Akaike Information Criterion. All results are presented with their 95% confidence intervals. Statistical tests were two-sided at the 5% level of significance. All the statistical analyses were done using R.2.3.0 statistical software [39].

## Results

### Frequencies of amplifications and co-amplifications

**Figure 1:**
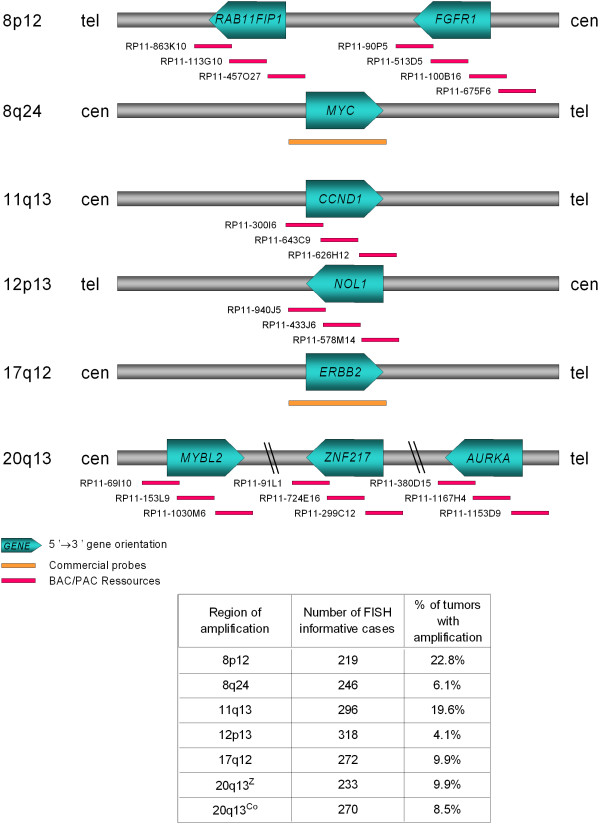
**Amplification regions and FISH probes**. Schematic representation of the six regions analyzed with probes used in FISH experiments and the main corresponding genes. The number of informative cases and the percentage (frequency) of amplifications of each analyzed region is given in the table.

Six regions of amplification were analyzed: 8p12 (amplified at least at one locus: *RAB11FIP1 *and/or *FGFR1*), 8q24 (*MYC*), 11q13 (*CCND1*), 12p13 (*NOL1*), 17q12 (*ERBB2*), and 20q13^Co ^(co-amplification of either two of three loci: *MYBL2, ZNF217 *and *AURKA*, or all three) regions. For the 20q13 region, in regard to the significant impact of *ZNF217 *amplification on disease evolution [29], we decided to distinguish the amplified subregion 20q13^Z ^centered on *ZNF217*. To assess the frequencies of the 8p12, 8q24, 11q13, 12p13, 17q12 and 20q13 amplifications we used FISH on TMA with specific probes. The number of interpretable cases varied between different FISH experiments (Figure 1). All TMA sections were only hybridized once. Reasons for non-informative results were: lack of tissue on the TMA, absence of unequivocal tumor cells, or non-interpretable hybridization data.

Among the informative cases, the frequency of amplification was 22.8% for 8p12, 6.1% for 8q24, 19.6% for 11q13, 4.1% for 12p13, 9.9% for 17q12, 9.9% for 20q13^Z ^and 8.5% for 20q13^Co ^regions (Figure 1). More in detail, the amplification frequencies of subregions centered on *RAB11FIP1 *and *FGFR1 *(8p12) as well as on *MYBL2, ZNF217 *and *AURKA *(20q13) loci are listed in Table 2. The highest amplication frequencies were obtained with *RAB11FIP1 *and *ZNF217 *subregions for 8p12 and 20q13, respectively.

**Figure 2:**
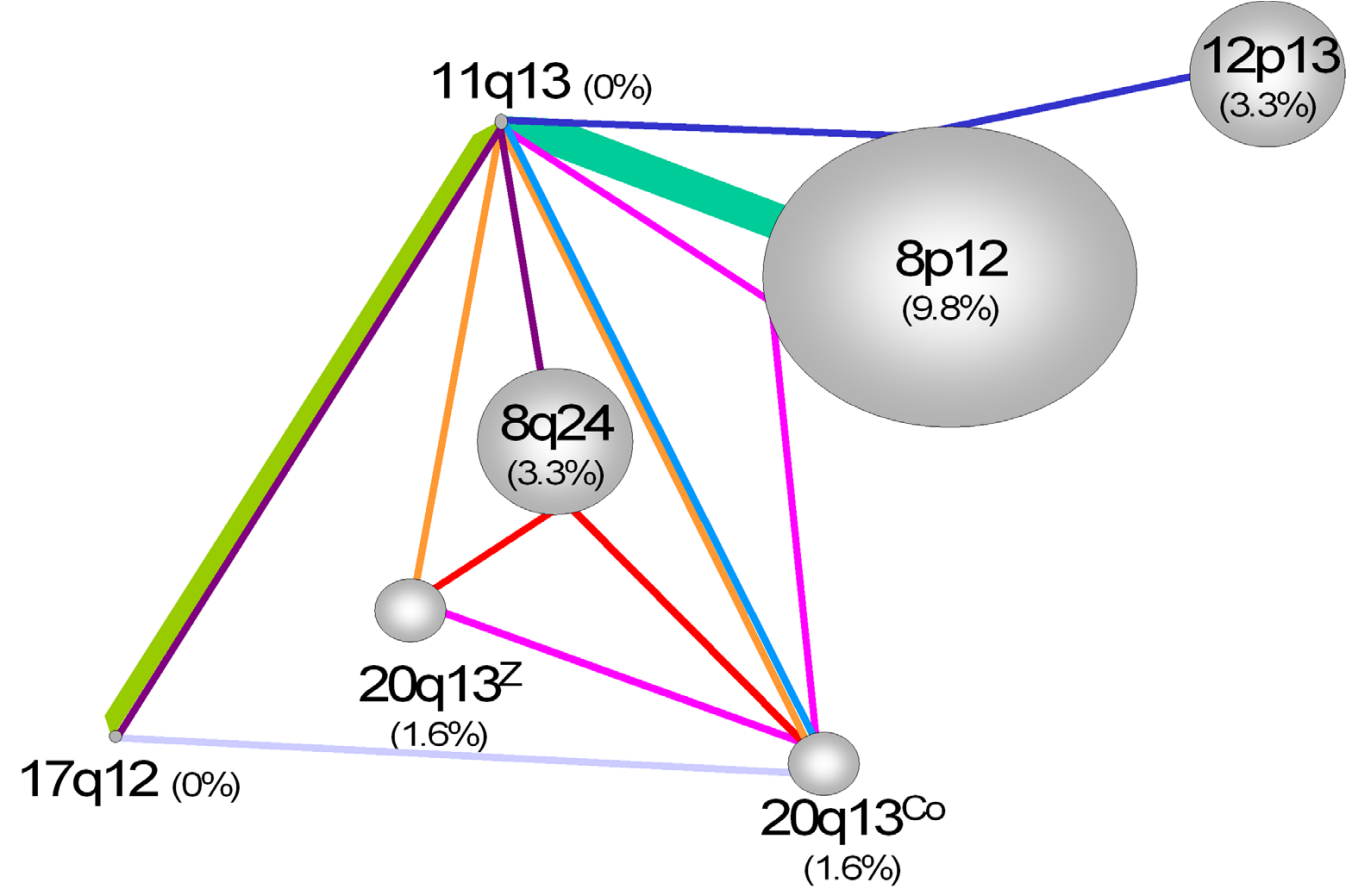
**Co-amplification network**. Schematic representation of frequencies of single amplifications and co-amplifications in 128 breast tumors samples. The size of spheres represents the frequency of region amplified as single. Lines of the same color represent co-amplification and the thickness of lines represents the frequency of the co-amplification: small: 1.6%, medium: 3.3%, large: 8.2%. For instance, the pink line linking 11q13, 8p12, 20q13^Co ^and 20q13^Z ^represents a co-amplification of all these regions found in 1.6% of informative cases (N = 128).

We then looked at the distribution of the frequency of amplifications and co-amplifications. Overall, only 128 cases were informative for all the regions analyzed. A total of 57.4% of cases showed no amplification. The frequency of single amplifications was 9.8% for 8p12, 3.3% for 8q24 and 12p13, 1.6% for 20q13^Co ^and 20q13^Z^. The 11q13 and 17q12 regions were never found amplified alone. The frequency of co-amplifications was 8.2% for 8p12/11q13, 3.3% for 11q13/17q12, 1.6% for 17q12/20q13^Co^, 11q13/20q13^Co^, 11q13/20q13^Z^/20q13^Co^, 8q24/11q13/17q12, 8q24/20q13^Z^/20q13^Co^, 8p12/11q13/12p13 and 8p12/11q13/20q13^Z^/20q13^Co ^(Figure 2). We also looked for associations between amplified regions. The 11q13/20q13^Co^, 12p13/20q13^Z ^and 8p12/11q13 co-amplifications were among the most strongly correlated [see Additional file 1].

### Correlation of amplified regions with histoclinical factors

We next examined the relation between amplifications and histoclinical factors [see Additional file 2]. We did not find any association between amplified regions and age, histological type and pathological tumor size. Amplification of 8q24, 12p13, 17q12 and 20q13^Co ^regions were correlated with high grade. Amplification of 8p12, 12p13 and 17q12 were correlated with a high proliferation index. Amplification of 8q24, 12p13, 17q12, and 20q13^Z ^were associated with ER and/or PR negativity. The 20q13^Co ^amplification was associated with axillary lymph node invasion.

### Correlation of amplified regions with clinical outcome

**Figure 3:**
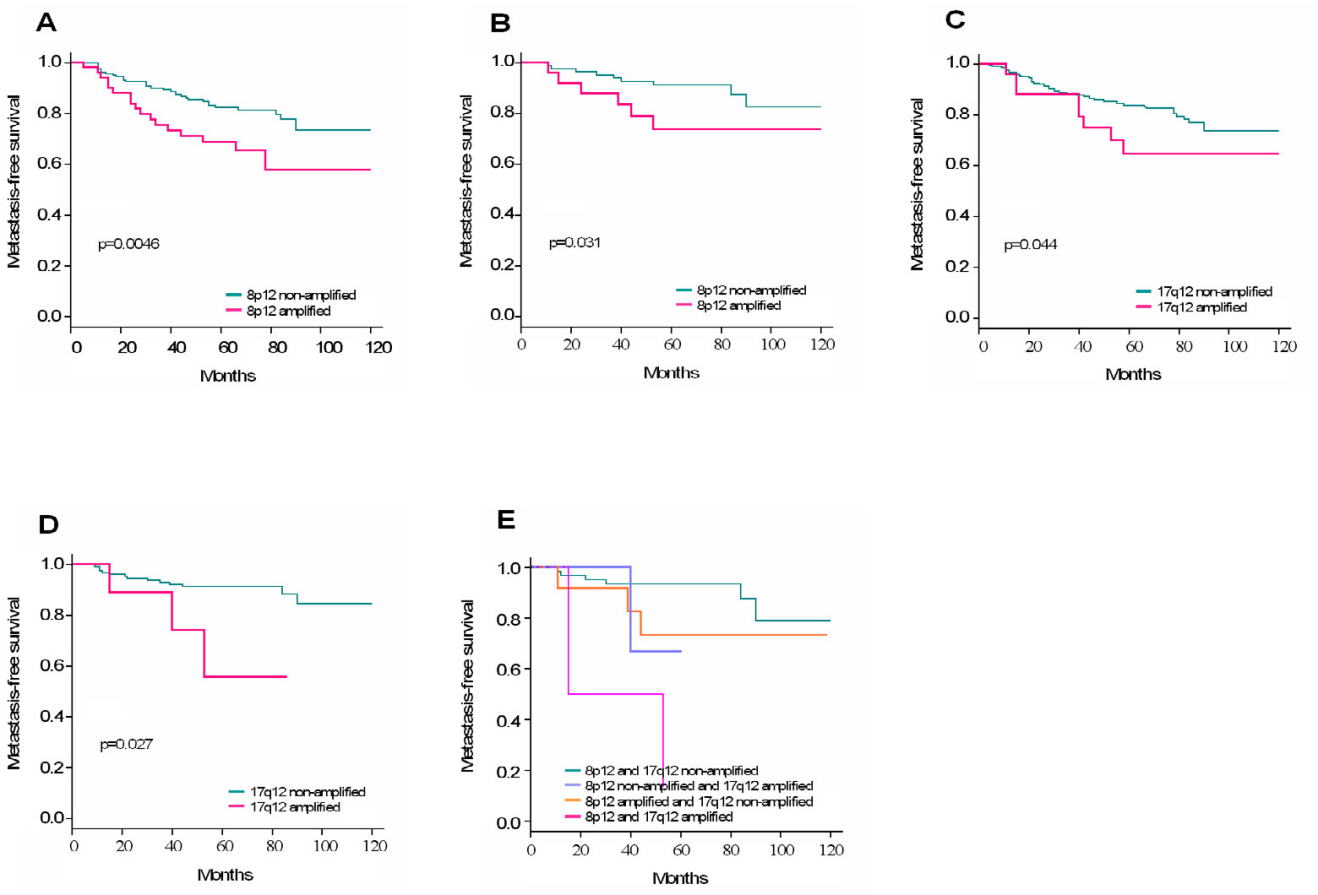
**Amplification status and associated metastasis-free survival in breast cancer**. **A**. Impact of the amplification of 8p12 region on MFS of the whole population (N = 219). **B**. Impact of the amplification of 8p12 region on MFS of N- patients (N = 114). **C**. Impact of the amplification of 17q12 region on MFS of the whole population (N = 272). **D**. Impact of the amplification of 17q12 region on MFS of N- patients (N = 140). **E**. Impact of the co-amplification of 8p12 and 17q12 regions on MFS of N- patients (N = 84). Kaplan-Meier curves illustrate MFS according to the status of amplification of different regions analyzed.

We then examined the impact of amplifications and co-amplifications on clinical outcome. We did univariate analyses to determine the impact of each individual amplified region on the MFS. The 8p12 and 17q12 amplifications but not the others were associated with clinical outcome (Table 3). Both were associated with decreased five-year MFS in the whole population and in N- patients (Figure 3A–D).To estimate the impact of co-amplification on MFS we did a multivariate analysis of significant amplifications in univariate analysis followed by a stepwise selection. We found that co-amplification of 8p12/17q12 was associated with a poor outcome in N- patients (Figure 3E).

We further evaluated the importance of the amplifications as prognosis markers. We did a Cox multivariate analysis of MFS. The values for amplification, grade, age, tumor size, peritumoral vascular invasion, ER, PR, and Ki67 were considered as categorical variables. Amplification status of 8p12 remained significant as well as Ki67 status according to the Akaike Information criterium when dichotomized amplified vs. non-amplified and <20 vs. ≥ 20 in N- patients (Table 4). The relative risk of recurrence was 2.52 for 8p12-amplified disease compared to non-8p12-amplified disease (p = 0.15).

### Correlation of amplified regions with molecular subtypes

Five main molecular subtypes (luminal A, luminal B, basal, ERBB2-overexpressing, and normal-like) have been identified by gene expression profiling of breast tumor samples using an intrinsic set of ~500 genes [40,41]. We determined the subtype of our samples by both mRNA and protein analyses (not shown). The 11q13 amplification was strongly correlated with the luminal A subtype (p value = 0.00029) and negatively correlated with the basal subtype (p value = 0.005633), whereas the 12p13 amplification was correlated with the basal subtype (p value = 0.020). As expected, the 17q12 amplification was correlated with the ERBB2 subtype (p value = 2.061e-06) (Table 5).

## Discussion

Several chromosomal regions are frequent targets for gene amplification in breast cancers. Oncogene activation and genomic instability associated with this process may play a role in tumor initiation and/or progression. Moreover, amplification may have prognostic and/or therapeutic significance for patients with breast cancer. However, few studies have looked at multiple amplifications and their potential correlations with tumor features and patient outcome [3,4].

Using FISH on TMA, we assessed the frequency of six amplifications, their potential association and their impact on clinical outcome. FISH technique is an easy and rapid method for amplification detection [42] and may provide prognostic information sometimes superior to other methods [43].

### Frequency of amplifications and correlations with prognosis

The most frequent amplification and co-amplification involved the 8p12 region. Several studies have shown that 8p12 is a common region of amplification and may harbor important breast cancer oncogenes [5,44-46]. This region, like other "hot spots" for gene amplification, may contain several genes that contribute to cell transformation. Because the exact 8p loci of significance are not known, we analyzed two subregions of 8p12 spanning many potentially relevant genes [5,44-46]. The two subregions covered genes that are amplified and most of them are overexpressed [5]. The frequency of 8p12 amplification in our breast tumor series was greater than the 10–15% commonly reported in literature, but similar to that recently reported by Garcia *et al*. (2005) [45]. Our results strengthen the idea that the 8p amplification, when all subregions are combined, may occur in a higher number of breast cancer than published so far. The 8p amplification was correlated with the amplification of 11q13 region: while 24.6% of tumors contained either amplified 8p12 region or 11q13 region, simultaneous amplification of both was seen in 10.8%. This result is in agreement with a previous study [47,48]. This suggests a coordinated mechanism of amplification and oncogene activation, and some kind of relation between proteins encoded by genes from the two regions. The 8p12 amplification tended to have aggressive tumor features such as high proliferative index and high SBR grade, but no association with histological subtype was found. With respect to prognosis, the 8p amplification was a significant predictor of reduced MFS. Multivariate analyses indicated that amplification of 8p12 added to the prognostic power of Ki67 status to define high risk N- patients. Our findings are consistent with the idea that 8p12 is a common region of amplification that may play a role in tumor behavior and/or pathogenesis. Previous analysis of 8p12 subregional amplifications in breast cancer [5] pointed to the amplified *FGFR1 *subregion as the best prognostic marker of bad disease evolution among other 8p amplifications. The identification of the best candidate drivers or the best therapeutic targets could refine the impact of the 8p12 amplification on survival and help determine which population should be targeted.

The *MYC *gene on 8q24 encodes a transcriptional regulator whose expression is strongly associated with cell proliferation. *MYC *amplification occurs in several types of cancers [49,50]. We found that the incidence of *MYC *amplification was in the range of those described previously [19]. About 13% of tumors contained either amplified *MYC *or *ERBB2*, whereas simultaneous amplification of both was seen in 1.9%. About 22% of tumors contained either amplified *MYC *or *CCND1*, whereas simultaneous amplification of both was found in 4.2%. *MYC *amplification was correlated with high grade and tended to be associated with high proliferation index, in agreement with previous studies [19]. Although the expression of the MYC protein is stimulated by estrogen and downregulated by tamoxifen in hormone-responsive breast tumors in vitro [51-54], we found that *MYC *amplification was correlated with the absence of estrogen receptor, which is in agreement with other studies [21,55] but contradictory to others [56,57]. No correlation was found with hormonotherapy (data not shown). With respect to prognosis, *MYC *amplification was not associated with MFS, neither in the whole population nor in the two different lymph node populations of patients. This is consistent with a previous study [58].

Amplification of the 11q13 region is a relatively frequent event in breast tumors [59]. This region harbors four distinct subregions of amplification, which can be amplified independently or together in different combinations [59,60]. Candidate genes have been suggested such as *CCND1, EMS1, and PAK1 *[60]. Amplification of *CCND1 *is within the most frequently amplified subregion and is found in two-thirds of all 11q13 amplifications. *CCND1 *overexpression promotes tumorigenesis in transgenic mice [61]. Amplification could promote sustained expression, which may cause the cell to cycle continuously. Amplification of *CCND1 *was found in 19.6% which is close to what is usually found in breast tumors. No association with histoclinical factors or survival was found, in disagreement with published data [22,62].

Amplification of 12p13 in breast cancers was first identified by Dib *et al*. (1994) [6] and characterized by comparative genomic hybridization by Yao *et al*. (2006) [7]. Overexpression of presumptive amplicon genes was found in medullary breast cancers [25]. Amplification of the short arm of chromosome 12, mostly as isochromosome, or as amplified 12p11-12 and 12p13 regions, is a hallmark of testicular germ cell tumors (TGCT) [63-65] and its overrepresentation is related to invasive growth of TGCT [66]. The 12p13 region harbors several candidate cancer genes. Human embryonic stem cell genes *NANOG, GDF3 *and *STELLA*, which are downregulated when cells commit differentiation, are expressed in TGCT and in breast cancers [67,68]. This suggests a role of these stem cell genes in carcinoma progression. Like CCND1, the CCND2 protein is involved in G1 phase of the cell cycle. *NOL1 *encodes the nucleolar protein P120, a proliferation-associated antigen that is temporally regulated during the cell cycle with an increase in protein expression at the G1/S transition. The choice of the *NOL1 *region for FISH was further suggested because its protein expression is associated with overall survival in node-negative breast cancers [69]. However, we did not find any association between amplification and survival. The 12p13 amplicon was correlated with 20q13^Z^, which may also be associated with proliferation.

*ERBB2 *amplification was found in 9.9% of tumors and was associated with high grade, absence of steroid receptors, overexpression of ERBB2 protein, and high proliferation index, which is an agreement with previous studies [17,70,71]. In univariate analysis, *ERBB2 *amplification was associated with MFS in the whole population and in N- patients. This is consistent with a poor outcome reported in several studies [43,71-73].

We found the 20q13 region amplified in 8.5–9.9% of tumors, which is consistent with frequencies previously reported in sporadic breast cancers [3,27-29] as well as in familial breast cancer [74]. The 20q13 amplified region was associated with 8q24, 11q13 and 12p13 amplifications. Co-amplification of 11q13/20q13 occurred in 1.6% of cases. The 20q13^Co ^amplification was associated with high grade and axillary lymph node invasion, in agreement with previous studies [27,29]. Amplification of 20q13^Z ^was associated with progesterone receptor negativity and with an accumulation of P53 in cells. Aberrant expression of 20q13 genes may be selected during breast cancer progression because it allows breast cells to overcome senescence and/or apoptosis. This is likely to be due to *ZNF217 *which promotes immortalization of human mammary epithelial cells [33] and plays a role in suppressing apoptosis [34]. Overexpression of other genes such as *AURKA *or *MYBL2*, which encode proteins involved in cell cycle regulation, could generate genomic instability. This accumulation of alterations could then lead to an increased sensitivity to chemotherapy and may explain the better prognosis for patients with amplified 20q13^Co ^region [29].

### Amplifications and molecular subtypes

We found that the 12p13 amplification was correlated with the basal subtype, which is in agreement with previous expression profiling data [25]. Moreover, the 12p13 amplification was correlated with high grade, absence of steroid receptors and high proliferation index, which are all features of the basal subtype. In contrast, the 11q13 (*CCND1*) amplification was negatively correlated with the basal subtype and strongly correlated with the luminal A subtype. This is consistent with a previous study [75] that found an inverse correlation between basal-like markers and *CCND1 *amplification.

## Conclusion

Our results show that regional amplification and co-amplification can be associated with pejorative evolution of breast cancer. Prognosis relevance applies for 8p12 and 17q12 amplifications analyzed as individual variable, and for 8p12/17q12 co-amplification. The 8p12 region has the most important impact on clinical outcome in two populations of patients: whole and axillary lymph node-negative. Therefore, 8p12 amplification could be used as a marker of adverse evolution in good prognosis breast cancer.

## Abbreviations

Amp: Amplification, BAC: Bacterial artificial chromosome, ER: Estrogen receptor, FISH: Fluorescence in situ hybridization, IHC: Immunohistochemistry, MFS: Metastasis-free survival, N-: axillary lymph node negative, N+: axillary lymph node positive, NS: Not significant, PR: Progesterone receptor, TGCT: Testicular germ cell tumors, TMA: Tissue microarray.

## Competing interests

The author(s) declare that they have no competing interests.

## Authors' contributions

AL, FS, CG, NC, FM, VGB carried out the FISH experiments, tissue microarray and immunohistochemistry were done by JG and read by ECJ, JJ. CZ, PF and BE did the statistical analyses. PV provided clinical data. AL, DB and MC drafted the manuscript. PV, DB and MC designed and coordinated the study. All authors read and approved the final manuscript.

## Pre-publication history

The pre-publication history for this paper can be accessed here:



## Supplementary Material

Additional file 1 Correlation between amplified regions analyzed by FISH.xls Click here for file

Additional file 2 Correlation between amplified regions determined by FISH and histoclinical factors.xls Click here for file

## Figures and Tables

**Table 1:** List of proteins tested by immunohistochemistry and characteristics of the corresponding antibodies.

Protein	Antibody	Origin	Clone	Dilution
Estrogen receptor	mmab	Novocastra Laboratories	6F11	1/60
Progesterone receptor	mmab	DakoCytomation	PgR 636	1/80
ERBB2	rpab	DakoCytomation	HercepTest	1/400
P53	mmab	Immunotech	DO-1	1/4
Ki67	mmab	DakoCytomation	MIB-1	1/100

mmab, mouse monoclonal antibody; rpab, rabbit polyclonal antibody

**Table 2:** 8p12 and 20q13 subregional amplification frequencies

Region of amplification	Gene regions	Number of FISH informative cases	% of tumors with amplification
8p12	*RAB11FIP1*	279	13.3%
	*FGFR1*	319	9.4%

20q13	*MYBL2*	265	5.7%
	*ZNF217*	233	9.9%
	*AURKA*	282	3.5%

**Table 3:** Cox univariate analyses of metastasis-free survival.

Region of amplification	Population			5-year M FS [95% CI]		Hazard ratio [95% CI]	*p*-value
			
	W hole	N +	N -	With amplification	Without amplification		
8p12	+			68.75 [56.73–83.3]	82.4 [76.6–88.7]	2.25 [1.28–3.93]	**0.0046**
8p12		+		63.2 [46.6–85.6]	72.6 [63–83.67]	1.87 [0.95–3.71]	0.071
8p12			+	73.77 [57.59–94.49]	91.33 [85.37–97.70]	2.98 [1.1–8.06]	**0.031**
8q24	+			78.6 [59.8–100]	82.61 [77.63–87.92]	0.825 [0.25–2.75]	0.75
8q24		+		75 [50.3–100]	74.53 [66.32–83.75]	0.728 [0.17–3.04]	0.66
8q24			+	83.3 [58.3–100]	89.55 [84.09–95.36]	0.825 [0.09–7.66]	0.87
11q13	+			75.93 [65.28–88.31]	83.95 [79.18–89]	1.46 [0.8–2.67]	0.22
11q13		+		75.72 [61.54–93.17]	74 [65.7–83.4]	0.962 [0.44–2.1]	0.92
11q13			+	76.02 [60.88–94.92]	91.72 [86.91–96.80]	2.46 [0.93–6.48]	0.069
12p13	+			91.67 [77.29–100]	82.23 [77.85–86.85]	0.427 [0.06–3.08]	0.4
12p13		+		100 [100-100]	72.14 [64.84–80.26]	*	1
12p13			+	83.3 [58.3–100]	91.28 [86.85–95.94]	2.46 [0.32–18.75]	0.39
17q12	+			64.7 [47.5–88.2]	83.92 [79.28–88.83]	2.09 [1.02–4.26]	**0.044**
17q12		+		68.2 [48.6–95.7]	75.37 [67.54–84.10]	1.24 [0.52–2.95]	0.63
17q12			+	55.6 [27.4–100]	91.25 [86.44–96.33]	4.2 [1.18–14.95]	**0.027**
20q13^Z^	+			85.4 [71.27–100]	79.43 [73.82–85.47]	0.57 [0.18–1.83]	0.35
20q13^Z^		+		90.91 [75.41–100]	63.98 [54.65–74.89]	0.186 [0.03–1.35]	0.097
20q13^Z^			+	79.5 [57.7–100]	93.05 [88.18–98.19]	2.58 [0.55–12.15]	0.23
20q13^Co^	+			90.43 [78.63–100]	79.80 [74.71–85.24]	0.364 [0.09–1.51]	0.16
20q13^Co^		+		87.84 [73.37–100]	66.80 [58.20–76.66]	0.276 [0.07–1.15]	0.076
20q13^Co^			+	100 [100-100]	90.40 [85.35–95.75]	*	1

*No event in the amplified group

**Table 4:** Cox multivariate analysis of metastasis free survival for patients without axillary lymph node invasion.

Variable	Coefficient value	Hazard ratio 95% CI	*p*-value
8p12	0.925		0.15
Non Amplified		1	
Amplified		2.52 (0.72–8.79)	
Ki67	1.29		0.055
<20		1	
≥20		3.63 (0.97–13.52)	

**Table 5:** Correlation between the amplification and molecular breast subtypes.

Region of amplification	Basal	Non Basal	*p*-value	ERBB2	Non ERBB2	*p*-value	Luminal A	Non Luminal A	*p*-value
						
	No. of patients (%)			No. of patients (%)			No. of patients (%)		
11q13									
*Amplification*	1 (5.5)	22 (45.8)	***p *= 0.006**	2 (25)	21 (36.2)	NS	19 (57.6)	4 (12.1)	***p *= 0.0003**
*No Amplification*	17 (94.5)	26 (54.2)		6 (75)	37 (63.8)		14 (42.4)	29 (87.9)	
12p13									
*Amplification*	5 (27.8)	2 (4.2)	***p *= 0.02**	1 (11.1)	6 (10.5)	NS	1 (3.1)	6 (17.6)	NS
*No Amplification*	13 (72.2)	46 (95.8)		8 (88.9)	51 (89.5)		31 (96.9)	28 (82.4)	
17q12									
*Amplification*	0 (0)	6 (17.6)	NS	5 (71.4)	1 (2.2)	***p *= 2.061e-06**	0 (0)	6 (20)	NS
*No Amplification*	19 (100)	28 (82.4)		2 (28.6)	45 (97.8)		23 (100)	24 (80)	p = 0.065

NS: Not significant
